# Epidemiological evaluation of cat health at a first-response animal shelter in Fukushima, following the Great East Japan Earthquakes of 2011

**DOI:** 10.1371/journal.pone.0174406

**Published:** 2017-03-30

**Authors:** Aki Tanaka, Philip H. Kass, Beatriz Martinez -Lopez, Shinichi Hayama

**Affiliations:** 1 Department of Population Health and Reproduction, University of California, Davis, California, United State of America; 2 Department of Wildlife Medicine, Nippon Veterinary and Life Science University, Musashino-shi, Tokyo, Japan; 3 Department of Medicine and Epidemiology, University of California, Davis, California, United State of America; University of South Carolina, UNITED STATES

## Abstract

The Great East Japan Earthquakes of March 11, 2011 caused immense harm to the community and subsequent nuclear accident in Fukushima Prefecture extended the damage. Local residents were forced to evacuated without pets and the left behind animals were rescued from the restricted zone one month later. Unplanned animal rescue and unregulated sheltering caused secondary damage to animals such as disease epidemics at impounded animal shelter. The purpose of this study was to retrospectively evaluate the incidence of upper respiratory infection (URI) and diarrhea in cats at the first response animal shelter in Fukushima, and investigate factors affecting the duration of disease and determinants of treatments performed. Eighty percent and 59% of impounded cats developed URI, 71% and 54% of cats developed diarrhea, and 91% and 83% of cats had at least one disease in 2011 and 2012, respectively. Uses of multiple drug administration (more than five drugs) was associated with prolonged URI and diarrhea. Multiple antibiotics, antihistamines, interferon, and steroids were associated with relapse of and prolonged URI. Developing a standardized treatment protocol for commonly observed diseases at Japanese animal shelters to prevent and control diseases, to promote animal welfare, and protect public health in the face of future disasters is overdue.

## Introduction

After the Great East Japan Earthquakes of March 11, 2011, a 20 km radius around the Fukushima Daiichi Nuclear Power Station was initially established as a restricted zone due to the radiation emergency (and has since been designated as an-evacuation directive lifted area). All residents within this area (over 110,000 people) were evacuated. The evacuation order was issued with no advance warning, and most local residents had no time to assemble their belongings to accompany them under the assumption that they would soon return. Consequently, their property, including pets, was forsaken for more than a month.

The Japanese government launched on April 27^th^, 2011 an animal rescue operation, and the first temporary shelter was established in Ihno, Fukushima prefecture. Most human shelters did not allow animals, so dogs and cats accompanying owners were either brought to the Ihno shelter or transferred to neighboring animal hospitals. The animal rescue operation originally focused on searching for companion animals in the restricted zone, and was incepted based upon pressure from pet owners forced to abandon their pets. The consequences of abandonment included: animal starvation, emaciation, and death; illegal entry to feed or reclaim animals with resultant exposure to unsafe radiation levels; uncontrolled animal reproduction; and harm to the area’s ecosystem.

The animal rescue operation in Fukushima took place for more than four years (and ended in December 31, 2015) due to the ongoing nuclear emergency at the power plant, while temporary animal shelters located in the disaster regions affected by the tsunami and earthquakes (Miyagi prefecture) closed mostly within six months. The Ihno shelter was initially established at a warehouse and was unsuitable for sheltering dogs and cats for a prolonged time; therefore, a second shelter was built in Miharu, Fukushima, and all animals were transferred there in January 2013. Because the animal rescue operation time was lengthy, it evolved through two phases. The initial chaotic phase suffered from lack of infrastructure, resources, and competent staff, exaggerating an already stressful situation. In the first seven months of the initial phase, there were no guidelines or protocols for managing animals at the shelter, different volunteer veterinarians engaged in the care of animals each day, and infectious diseases were extremely common. The second phase was far more stable: most of the animals were tamed, socialized, and experienced a low incidence of disease.

The primary health issue reported for cats at animal shelters was upper respiratory infection (URI) [1–4]. The inherent properties of animal shelters, which include a constant influx of animals without medical backgrounds or vaccination histories, overcrowding, lack of quarantine or isolation facilities, poor hygiene, loud noise, housing with dogs, confinement to cages, and extremely stressful environments, act as risk factors for endemic infectious diseases[1, 4–6]. Although the prevalence of causative agents is shelter-specific[7–9], the majority of URI is caused by feline herpesvirus-1 (FHV-1), feline calicivirus (FCV), *Bordetella bronchiseptica*, *Chlamydophila felis*, and *Mycoplasma* species[4, 10, 11]. Apparently healthy cats can be carriers for all of these agents; this and the risk that shelters pose make control of URI extremely difficult in the absence of standardized protocols for disease prevention and management.

Diarrhea is also a common disease in sheltered cats; the causes are multifactorial, including stress, diet, and viral, protozoal, bacterial, or helminthic infection[11, 12]. It also shares common risk factors with URI in shelters, including crowding, stress, poor hygiene practice, and asymptomatic shedding cats[5, 12]. Although causative agents for URI and diarrhea and risk factors for disease development are known, shelters do not necessarily have the resources to establish definitive diagnoses, and symptomatically treating cats without validated guidelines for shelter management will not necessarily resolve clinical illness or prevent the spread of infectious disease if the risk factors go uncontrolled.

In this research, a retrospective epidemiological evaluation of sheltered cats was performed to investigate feline health status and treatment protocols during the first and second years at the Ihno, Japan shelter. The incidences of URI and diarrhea were compared between the first and second years, and related to factors predictive of disease duration and frequency, including choice of treatment options.

## Materials and methods

### Study population

A retrospective cohort study was performed at a temporary disaster shelter in Ihno, Fukushima prefecture, Japan. Between April 27^th^, 2011 to December 31st, 2012, 189 cats brought by animal control officers from the restricted area to the temporary disaster shelter as part of an animal rescue operation following the Great East Japan Earthquakes of 2011 were included. Ninety five cats were impounded during 2011, and 94 cats were impounded during 2012. Animals were held at this shelter until they were transferred to a newly built shelter in Miharu, Fukushima prefecture in 2013. Euthanasia was not performed unless animals suffered from severe illness. The Ihno shelter remained open from April 27^th^, 2011 until December 31th, 2012, and the Miharu shelter opened on October 1^st^, 2012 and operated until December 31st, 2015.

### Syndrome/disease definition and clinical description

In 2011 different volunteer veterinarians managed animals at the shelter and examined each cat daily until a full-time veterinarian was hired in January, 2012. Any signs of conjunctivitis, anterior uveitis, keratitis, serous or purulent ocular discharge, nasal discharge (characterized as serous or purulent), sneezing, gingivitis, oral vesicles, or faucitis were recorded and diagnosed as upper respiratory infection (URI). Any clinical signs consistent with diarrhea (characterized as frequent loose or liquid bowel movements) were recorded and diagnosed as diarrhea. All animals with these clinical signs present at admission were excluded from the study. The date the animals developed their clinical disease following shelter entrance, the date the sign(s) resolved, and any drugs administered were recorded.

Data collection: Sex, intake date, vaccination date, location of capture, health status (any abnormal signs observed on daily rounds were recorded in the medical record by the veterinarian), duration and frequency of URI and diarrhea, and administered drugs were recorded.

### Statistical analysis

Kaplan-Meier survival analysis and the log-rank test were used to determine whether the impounded year (2011 vs 2012) was associated with the incidence of URI and diarrhea development. Cox proportional hazards regression models were used to assess the association between potential risk factors (number of drugs administered, days on medication, use of corticosteroids and interferon, number of different antibiotics used, and other drugs used) and duration of the diseases (URI and diarrhea). Incidence rate ratios (IRR) and 95% confidence intervals (CI) were calculated. Interactions between variables were assessed for improvement in model fit by performing likelihood ratio tests contrasting nested models. Multilevel mixed-effects Poisson regression models were used to assess the association between the potential risk factors and the count of temporally independent URI events (each event was counted individually when the complete resolution of the previous symptoms and development of new URI symptoms was two or more weeks apart); results are reported as IRR and 95% CI. Logistic regression was used to determine whether relapse of URI was related to the same potential risk factors; results are reported as odds ratios (OR) and 95% CI. Variables with p<0.20 will initially be included in a multivariable model, and Cox proportional hazards regression, multilevel mixed-effects Poisson regression models, and logistic regression with backward elimination will be performed to identify a model containing variables whose coefficients significantly differ from 0. Interactions between variables will be checked by performing likelihood ratio tests contrasting models with and without the interactions. Any clinically important and/or statistically significant interactions will be included in the model.

Commercially available statistical software (Stata/IC 13.1, StataCorp LP, College Station, Texas, SA) was used in all analyses. For statistical estimation and inferences, two-sided hypotheses and tests were used with a 5% significance level.

## Results

### Descriptive

Characteristics of the admitted cats are described in Table 1. One hundred and forty-eight cats were rescued from the restricted zone by the prefecture government in 2011; of these, 95 (52 females, 36 males, and seven of unknown sex) were admitted to the Ihno shelter in 2011, and 53 were transferred to animal hospitals or government shelters in other prefectures. In 2012, 97 cats were rescued from the restricted zone, and 94 (47 females, 46 males, and one of unknown sex) were admitted to the Ihno shelter. There was one kitten admitted to the shelter in 2011, but all other cats admitted during the study period were adults estimated to be older than one year. All cats received an inactivated vaccine containing feline rhinotracheitis virus, FCV, and panleucopenia virus (Fel-O-Vax 3, Boehringer Ingelheim Vetmedica Inc., St. Joseph, MO, USA) or inactivated feline rhinotracheitis virus, FCV, panleucopenia virus, *Chlamydophila felis*, and feline leukopenia virus (Fel-O-Vax 5, Boehringer Ingelheim Vetmedica Inc., St. Joseph, MO, USA). Median time from intake until first vaccination was 2 (range = 1–67) days and the median time until the second vaccination was 81 (range = 3–117) days in 2011. In 2012, all cats were vaccinated within 48 hours of intake with the same inactivated vaccines, but no records were kept for second vaccinations. All cats were screened for intestinal parasites by fecal flotation, and multiple intestinal parasites were identified including tapeworms, roundworms, and coccidia. Hematuria was observed in six and nine cats in 2011 and 2012, respectively. Parturition was observed following shelter admission in approximately 60% of female cats in 2012.

**Table 1:** Characteristics of cats admitted to the Ihno, Japan animal shelter in 2011 and 2012 and observed characteristics of the disease during their stay at the shelter.

	2011		2012	
	(n = 95)		(n = 94)	
**Variables**	**Number of cats**	**Percentage**	**Number of cats**	**Percentage**
**Sex**	52	54.7	47	50
**Female**	36	39.7	46	48.9
**Male**	7	7.4	1	1.1
**Unknown**	32	33.7	39	41.5
**Roundworm**	8	8.4	2	2.1
**Coccidia**	10	10.5	Unknown	Unknown
**Sex**	6	6.3	9	9.6
**Parturition after admission**	0	0	28	59.6
**Number of diarrhea**	67	70.5	51	54.3
**Number of URI**	79	83.2	55	58.5
**Number of cats with both diarrhea and URI**	50	52.6	28	29.8
**Number of cats with either diarrhea or URI**	91	95.8	75	79.8
**Recurrence proportion of URI**	26/79	32.9	15/74	20.3
	2011		2012	
	(n = 95)		(n = 94)	
**Variables**	**Days**	**Range**	**Days**	**Range**
**Median duration of URI symptom**	15.5	1–174	8	1–110
**Median duration of diarrhea symptom**	3	1–22	4	1–16
**Median duration to occurrence of URI**	17.5	1–136	25	1–112
**Median duration to occurrence of diarrhea**	30	1–136	59	1–189

### Infectious disease

**Fig 1:**
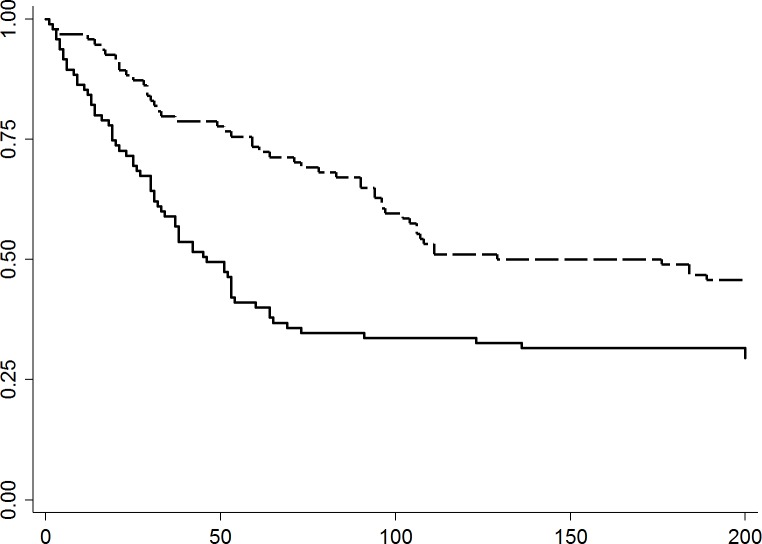
Kaplan-Meier survival curves of days to development of diarrhea among cats in 2011 (n = 95; solid line) or 2012 (n = 94; dashed line) during their shelter stay. **Curves are significantly different (p = 0.001).** X-axis; Days at shelter. Y-axis; Probability free of diarrhea. Dashed line; year 2012. Solid line; year 2011.

Incident diarrhea was observed in approximately 71% of cats in 2011, and 54% of the cats in 2012. The 2011 incidence of diarrhea in cats was highest between one and two weeks after entering the shelter, with up to nine cases per week, and remained high until eight weeks after admission (except for week seven), declining to one new case per week by the 12^th^ week of shelter residence. In 2012 the first peak was observed in Week 5, with up to six new cases per week, and declined to one new case per week the following week (Fig 1). The median days (range) to develop clinical signs of diarrhea following intake was 30 (1–136) days in 2011 and 59 (1–189) days in 2012, respectively. The median duration (range) the cats exhibited diarrheic signs was 3 (1–22) days and 4 (1–16) days in 2011 and 2012, respectively (Table 1).

**Fig 2:**
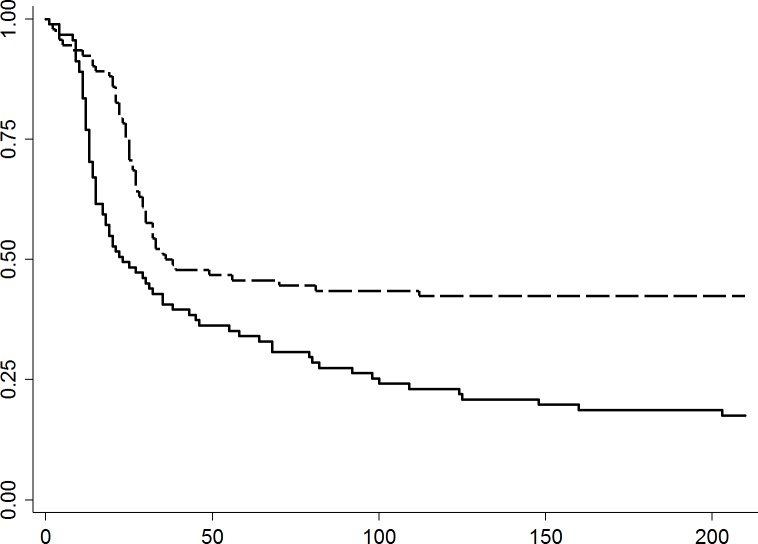
Kaplan-Meier survival curves of days to development of URI among cats in 2011 (n = 95; solid line) or 2012 (n = 94; dashed line) during their shelter stay. **Curves are significantly different (p = 0.001).** X-axis; Days at shelter. Y-axis; Probability free of URI. Dashed line; year 2012. Solid line; year 2011.

Upper respiratory infections were observed in more than 95% and approximately 80% of the cats in 2011 and 2012, respectively. The highest incidence of URI was seen between Weeks 2 and 3, with a maximum of 27 new cases in one week, decreased to one new case by Week 6, and remained in a low incidence for the remainder of 2011. In 2012 the peak occurred between Weeks 4 and 5, with a maximum of 18 new cases in one week, and decreased to one new case per week by Week 7 (Fig 2). The median days (range) to development of clinical signs of URI following intake date was 17.5 (1–136) and 25 (1–112) days. The median duration (range) the cats presented the URI signs was 15.5 (1–174) days and 8 (1–110) days in 2011 and 2012, respectively. Approximately 26 of 79 cats that developed URI (33%) and 15 of 74 (20%) cats that developed URI in 2011 and 2012, respectively had relapse of URI events (Table 1).

Approximately 96% of cats (91 of 95) either showed signs of URI or diarrhea in 2011, and approximately 80% of cats (75 of 94) in 2012. Approximately 63% of cats (60 of 95) in 2011 and approximately 30% of cats (28 of 94) in 2012 had both URI and diarrhea (Table1). In 2011, the odds ratio that cats acquired both URI and diarrhea was higher than 2012 (OR = 1.91, 95% CI = 1.34–2.73, p <0.001; and OR = 1.83, 95% CI = 1.27–2.64, p = 0.001, respectively), and having diarrhea was associated with subsequent development of URI (OR = 3.59, 95% CI = 2.41–5.35, p < 0.001).

### Medications

**Fig 3:**
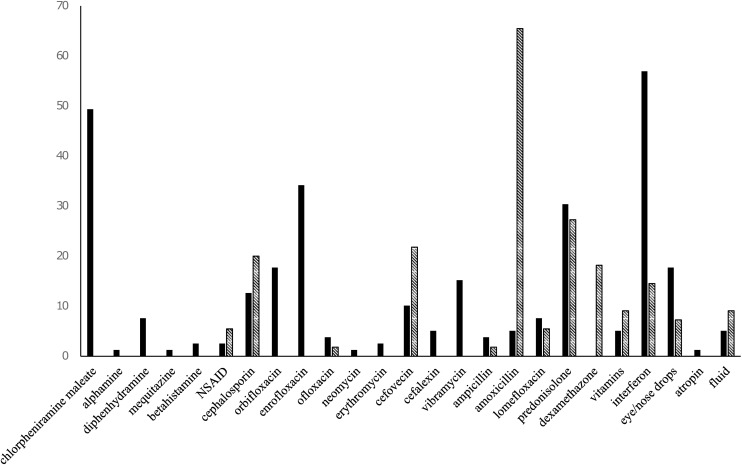
Medications administered to cats to treat at Ihno shelter for URI in 2011 (n = 79) and 2012 (n = 55). Y-axis; Percentage of cats. Solid bar; year 2011. Dashed bar; 2012.

**Fig 4:**
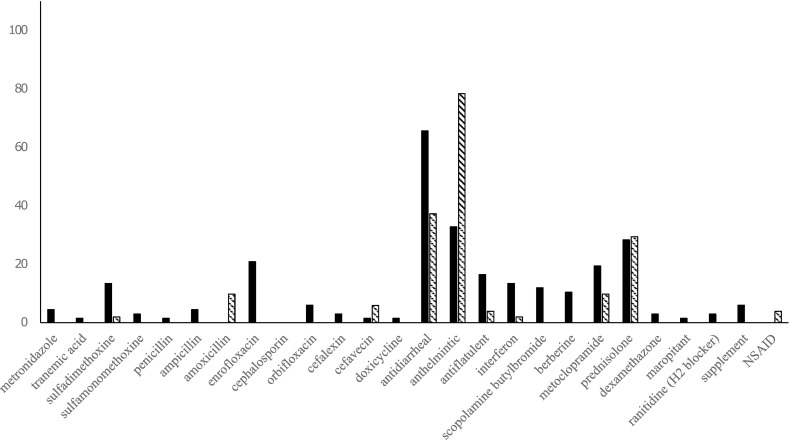
Medications administered to cats to treat diarrhea at Ihno shelter in 2011 (n = 67) and 2012 (n = 51). Y-axis; Percentage of cats. Solid bar; year 2011. Dashed bar; 2012.

Medications administered to cats to treat URI and diarrhea are summarized in Figs 3 and 4. Corticosteroids were administered to approximately 30% of cats with URI and more than 40% of cats with diarrhea in 2011, and more than 40% of URI cases and approximately 30% of diarrheic cases were given corticosteroids in 2012.

Antibiotics were given to 67.4% of cats (64 of 95) in 2011, and more than 40% received more than three different antibiotics. The median number (range) of different drugs administered per cat in 2011 was 6 (1–16); half the cats were administered five to eight drugs, and 20% received more than nine drugs. In 2012, 75% of cats were given less than four drugs.

Interferon was given to approximately 66% of the cats (52 of 79) to treat URI in 2011. The median duration (range) of medication administration per cat in 2011 was 11 (1–52) days, with approximately 30% were medicated for more than three weeks; while in 2012 the median duration (range) of medication administration was 3 (1–88) days; most were treated less than two weeks.

Interferon (IRR = 0.48, 95% CI = 0.21–1.1, p = 0.080) and antihistamine (IRR = 0.45, 95% CI = 0.30–0.68, p < 0.001) were associated with prolonged duration of URI, as were the number of antibiotics and the number of drugs administered (Table 2). Metronidazole (IRR = 0.29, 95% CI = 0.09–0.96, p = 0.043), enrofloxacin (IRR = 0.55, 95% CI = 0.30–1.00, p = 0.051), scopolamine butylbromide (IRR = 0.38, 95% CI = 0.18–0.80, p = 0.011), berberine (IRR = 0.44, 95% CI = 0.20–0.95, p = 0.037) were associated with longer duration of diarrhea, as was the number of drugs administered (Table 3). Factors associated with the frequency of URI included the number of drugs, number of antibiotics, steroids, antihistamines, interferon, and days medicated. After controlling for intake years (2011 and 2012), antihistamines and steroids remained associated with an increased frequency of URI (Table 4). Medications associated with relapse of URI were steroids (OR = 3.10, 95% CI = 1.44–6.68, p = 0.004), antihistamines (OR = 2.55, 95% CI = 1.19–5.49, p = 0.016), chlorpheniramine (OR = 4.32, 95% CI = 1.94–9.60, p < 0.001), enrofloxacin (OR = 2.31, 95% CI = 0.96–5.56, p = 0.063), cefovecin (OR = 2.65, 95% CI = 1.00–6.97, p = 0.049), prednisone (OR = 2.76, 95% CI = 1.11–6.82, p = 0.028), vitamins/fluids (OR = 7.71, 95% CI = 1.49–40.06, p = 0.015), and the number of drugs (when the number of administered drugs was nine or more, there was a greater than eight-fold higher odds of relapse of URI (OR = 8.59, 95% CI = 2.65–27.82, p < 0.001) (Table 5).

**Table 2:** Univariable and multivariable Cox proportional hazards model for risk factors associated with duration of the disease signs for URI syndrome.

Univariable models	IRR	95% CI	p-value
**Intake year**			
**2012 (n = 94)**	1.00		
**2011 (n = 95)**	0.5	0.34–0.74	0.001
**Interferon (n = 53)**	0.48	0.21–1.09	0.08
**Number of antibiotics (n = 101)**			
**1 (n = 44)**	1.00		
**2 (n = 29)**	0.38	0.23–0.64	<0.001
**3 or more (n = 28)**	0.25	0.15–0.44	<0.001
**Number of drugs (n = 105)**			
**1 to 4 (n = 53)**	1.00		
**5 to 8 (n = 34)**	0.38	0.24–0.61	<0.001
**9 or more (n = 18)**	0.20	0.11–0.39	<0.001
**Days under medication (n = 93)**			
**1 week (n = 56)**	1.00		
**2 weeks (n = 19)**	0.37	0.22–0.65	<0.001
**3 weeks (n = 9)**	0.24	0.10–0.54	0.001
**4 weeks (n = 9)**	0.25	0.12–0.53	<0.001
**Antihistamine (n = 113)**	0.45	0.30–0.68	<0.001
**Chlorpheniramine (n = 113)**	0.36	0.23–0.56	<0.001
**Multivariable model (n = 89)**			
**Number of antibiotics 1**	1.00		
**2**	0.61	0.34–1.11	0.106
**3 or more**	0.44	0.24–0.80	0.007
**Days under medication 1 week**	1.00		
**2 weeks**	0.44	0.24–0.81	0.009
**3 weeks**	0.30	0.13–0.73	0.008
**4 weeks**	0.34	0.15–0.78	0.011

**Table 3:** Univariable Cox proportional hazards model for risk factors associated with duration of the disease signs for diarrhea.

Univariable models	IRR	95% CI	p-value
**Intake year 2011 (n = 95)**	1.83	1.27–2.64	0.001
**Number of drugs (n = 102)**			
**1 to 4 (n = 54)**	1.00		
**5 to 8 (n = 32)**	1.16	0.74–1.80	0.517
**9 or more (n = 16)**	0.57	0.32–0.99	0.049
**Metronidazole (n = 118)**	0.29	0.087–0.96	0.043
**Enrofloxacin (n = 113)**	0.55	0.30–1.00	0.051
**Scopolamine butylbromide (n = 113)**	0.38	0.18–0.80	0.011
**Berberine (n = 113)**	0.44	0.20–0.95	0.037

**Table 4:** Univariable and multivariable Poisson regression model and incidence rate ratio (IRR) of the frequency of URI.

Univariable models	IRR	95% CI	p-value
Intake year 2011(n = 189)	4.00	3.00–5.40	<0.001
Parturition (n = 99)	0.42	0.26–0.68	<0.001
Number of drugs (n = 153)			
1 to 4 (n = 92)	1.00		
5 to 8 (n = 42)	2.64	1.86–3.75	<0.001
9 or more (n = 19)	5.41	3.67–7.97	<0.001
Number of antibiotics (n = 122)			
1 (n = 59)	1.00		
2 (n = 33)	1.22	0.84–1.76	0.299
3 or more (n = 30)	2.33	1.61–3.40	<0.001
Steroids (n = 189)	1.74	1.30–2.34	<0.001
Antihistamine (n = 175)	4.24	1.15–1.74	<0.001
Interferon (n = 189)	3.14	2.35–4.20	<0.001
Days under medication (n = 122)			
1 week (n = 79)	1.00		
2 weeks (n = 23)	2.51	1.69–3.74	<0.001
3 weeks (n = 9)	3.01	1.74–5.22	<0.001
4 weeks or more (n = 11)	3.03	1.72–5.33	<0.001
Multivariable model (n = 175)			
Intake year (2011)	3.81	2.59–5.62	<0.001
Antihistamine	1.46	0.99–2.16	0.058
Steroids	1.68	1.25–2.27	0.002

**Table 5:** Univariable and multivariable logistic regression analysis of factors associated with relapse of URI.

Univariable models	OR	95% CI	p-value
Number of drugs (n = 118)			
1 to 4 (n = 61)	1.00		
5 to 8 (n = 38)	1.25	0.50–3.10	0.63
9 or more (n = 19)	8.59	2.65–27.82	<0.001
Steroids (n = 133)	3.10	1.44–6.68	0.004
Antihistamine (n = 133)	2.55	1.19–5.49	0.016
Chlorpheniramine (n = 113)	4.32	1.94–9.61	<0.001
Enrofloxacin (n = 113)	2.31	0.96–5.56	0.063
Cefovecin (n = 113)	2.65	1.00–6.97	0.049
Prednisone (n = 133)	2.76	1.11–6.83	0.028
Vitamins/Fluids (n = 113)	7.71	1.49–40.05	0.015
Multivariable model (n = 133)			
Steroids	3.16	1.44–6.95	0.004
Antihistamine	2.61	1.18–5.79	0.018

## Discussion

Animal rescue operations were launched amidst a worsening chaotic situation when animal welfare was not a high priority; sheltering was hence established in a disorganized manner by inadequately trained personnel in 2011. Lack of established standardized procedures was manifest by the rather inconsistent therapeutic approaches for diseased cats, consequently leading to more frequent and longer duration communicable diseases. Different volunteer veterinarians worked every day, with no uniformity or consistency in treatments even on the same cats. Twenty-four different medications were used for treating URI in 2011, including five types of antihistamines, 13 types of antibiotics, two kinds of steroids, and others. Medicating with more than five drugs in the same patient was associated with longer duration of clinical signs, more frequent incidence, and relapse of URI.

The most common causative agents responsible for URI in shelters are FHV-1, FCV, *Mycoplasma* species, *Bordetella bronchiseptica*, and *Chlamydophila felis*[4, 10, 11]. Allergies are not commonly recognized as major contributors to URI clinical signs in shelters; however, more than 60% of the cats were treated with antihistamines, the majority of them with chlorpheniramine. Antihistamine treatment was associated with longer duration of the disease, higher incidence, and relapse of URI. Even after controlling for year, antihistamine use remained associated with more frequent disease incidence. The use of antihistamines in cats at the shelter to treat URI is not typically considered to be an appropriate treatment. Known risk factors for URI at shelters include stress, overcrowding, cats being housed close to dogs, cats being moved from cage to cage, poor hygiene, and housing styles[3, 8, 13–15]. Antihistamines for cats are typically reserved for treating allergic or atopic dermatitis[16, 17], rather than URI. For these reasons, antihistamines to treat URI for cats might not be experienced, especially in shelter settings.

Corticosteroids were administered to 30% of the cats with URI in this study. Cats at shelters are already under the stress of relocation, cage confinement, crowding, and possible immunosuppression, so administration of corticosteroids could exacerbate stress and suppress immune response at the very time that cats are exposed to communicable diseases[18]. Evidence shows that corticosteroid administration induces FHV-1 activation[14], and both contributing to initiation and complication of the clinical signs and disease course[19]. In the present study, the dosage of corticosteroid was unknown; however, any administration was associated with URI incidence, recurrence, and relapse of the upper respiratory infection syndrome. Although evidence supports the efficacy of corticosteroids for chronic stomatitis in cats[20, 21], it is still advisable to avoid using corticosteroids in cats that are highly stressed and possibly immunocompromised at shelters.

Interferon was administered to approximately 66% of cats with URI in this study. Although interferon has shown anti-FHV-1 activity *in vitro*[19], there is no evidence to date supporting its effectiveness for treatment of clinical signs of URI. In this study interferon administration was associated with more frequent URI incidence and longer duration of the disease. Interferon was effective for treatment of chronic gingivostomatitis in one report[22], and has been anecdotally reported to lead to clinical improvement for virulent systemic FCV disease[23]. The cats in the present study did not show severe symptoms applicable to these diseases, and virulent systemic FCV disease has not been reported in Japan to date. Interferon is an expensive drug (approximately $100 per one dose) and requires multiple injections, so is unlikely to be recommended to use when its efficacy has not been fully proven for typical URI for shelter cats.

A wide variety of antibiotics were used to treat URI in the Ihno shelter, including fluoroquinolones (orbifloxacin, enrofloxacin, ofloxacin, lomefloxacin), β-lactams (cephalosporin, cefalexin, ampicillin), neomycin, cefovecin, amoxicillin, erythromycin, and doxycycline. Studies indicate that doxycycline, pradofloxacin, and amoxicillin were effective for clinical resolution of URI[24, 25], and doxycycline and amoxicillin were more effective than cefovecin in treating clinical signs[26]. The most common first choices of antibiotic used in United States shelters are doxycycline (52%), amocixillin/clavulanic acid (33%), and azithromycin (7%)[27]. Multiple agents are involved in URI, and each causative agent has different susceptibility to the antibiotics. *Bordetella*. *bronchiseptica* isolates are susceptible to doxycycline and amoxicillin, but resistant to cefovecin, cephalosporin, and ampicillin[26, 28]. *Mycoplasma* species, *Bordetella bronchiseptica*, and *Chlamydophila felis* are resistant to β-lactams[29]. Enrofloxacin and doxycycline were effective in improving clinical signs of *Chlamydophila felis* infection[30]. In this study, enrofloxacin and cefovecin were associated with relapse of clinical disease. Performing cultures and determining antibiotic susceptibilities before treating URI in shelters is impractical, and even identifying the causative agent(s) in each patient is infeasible in shelter settings. However, inconsistent choice of antibiotic is medically contraindicated and potentially ineffective within individuals, and using two or more antibiotics was associated with prolonged clinical signs and frequent recurrence of the disease in this study.

The altruistic veterinarians eager to volunteer to care for the impounded cats had no standardized protocol at the shelter to adhere to, and so elected to use their own protocols to medically treat clinically ill animals. This situation led to cats receiving many different medications for treating URI, leading to longer illnesses, relapses, and higher incidence. Consistent antibiotic treatment for URI is recommended to continue for at least 21 days to eliminate pathogens[30]. In this study, cats medicated for more than one week experienced prolonged signs and more frequent disease occurrence compared to cats under medication for less than one week, which may be due to inconsistent drug therapy.

Diarrhea was commonly observed in the cats, with 70.5% affected following shelter admission in 2011, and 54% in 2012. Twenty-three different drugs were administered to treat diarrhea, including tranexamic acid, antibiotics, antidiarrheals, anthelmintics, antiflatulents, interferon, antimuscarinics, anticholinergics, berberine, metoclopramide, corticosteroids, H2 blockers, non-steroidal anti-inflammatories, and maropitant. Twelve different antibiotics were used, including nitroimidazole (metronidazole), sulfonamides (sulfadimethoxine, sufamonomethoxine), penicillins (penicillin, amoxicillin), β-lactams (ampicillin, cephalosporin, and cefalexin), fluoroquinolones (enrofloxacin, orbifloxacin), doxycycline, and cefavecin. Diarrhea in cats has multifactorial causes, include change of diet, stress, and infections with enteropathonegic viruses, parasites, protozoa, and bacteria[5, 12]. Not all the diarrheic cats were examined for enteropathogens in this study, but those known to be causal in cats include *Cryptosporidium* spp, *Cystoisospora* spp, *Giardia* spp, *Clostridium perfringens*, *Salmonella* spp, *Campylobacter jejuni*, ascarids, hookworms, astovirus, feline corona virus, *Toxoplasma gondii*, feline panleukopenia virus, FCV, and *Spirometra* spp[12, 31, 32]. *Salmonella*, *Campylobacter*, *Giardia*, *Cryptosporidium*, hookworms, and ascarids are pathogens with zoonotic potential; however, these enteropathogens can also be identified in normal feces[12, 31, 33]. The majority of bacterial enteropathogens are associated with self-limiting disease, and injudicious administration of antibiotics could be more harmful than beneficial[33].

In this study, using five or more drugs for treatment, and use of metronidazole, enrofloxacin, scopolamine butylbromide, and berberine were associated with longer duration of the disease. Antibiotic therapy can potentially alter microbiota, cause gut bacterial overgrowth[34], and deregulate defense against viruses. One study revealed that fecal bacteria isolated from sheltered cats were more frequently resistant than owned cats[35]. Multidrug-resistant salmonellosis to humans from cats has been reported in the U.S., where one of the foci of transmission was an animal shelter[36]. In that study’s shelter, diarrheic cats were constantly treated with antibiotics, and *Salmonella* isolates were found to be resistant to ampicillin, chloramphenicol, streptomycin, sulfamethozole, and tetracycline[36]. The Ihno shelter was a prime candidate for harboring antibiotic resistant pathogens considering the amount of antibiotics used for treating cats with both diarrhea and URI.

Patient resolution for both diarrhea and URI can occur in the absence of medical intervention, and the actual treatments performed may ostensibly have played a negligible positive role in improvement of the health of cats at the Ihno shelter. Administration of corticosteroids to cats at shelters is arguably contradicted not only because they are already stressed, but also because they tend to asymptomatically harbor FHV-1 and other viral pathogens. Interferon and antihistamines are ineffective in treating URI. Overall, indiscriminately treating the cats was ineffective, and reducing environmental stress in addition to development of and adherence to a standardized treatment protocol is by far a more important management strategy for controlling disease outbreaks. The mean number of days the cats had been symptomatic for both URI and diarrhea, and the incidence of both, was lower in 2012 compared to 2011. This may be related to the stationing of a full-time veterinarian in the later year, and treatment protocols were applied consistently (although not specifically standardized for a shelter setting). Although the vaccination and hygiene practices were sub-optimal, it was fortunate that there was no outbreak of feline panleukopenia infection at the Ihno shelter.

In the present study, the incidence proportion of diarrhea and URI in cats at Ihno shelter was high. High prevalences of diarrhea and URI were also common at other shelters due to stress, dietary changes and immunodeficiency[12, 37] [4, 7, 38, 39]. However, there is no evidence to date that shows that the diseases in this shelter were associated with radiation from the nuclear accident in Fukushima. The incidence of both diseases declined from 2011 to 2012. The possible reasons for this included: infrastructures were restored, environmental conditions in the affected area improved, there was less stress for both shelter staff and cats, there were more trained personnel for better care of cats, and housing conditions improved at the shelter. Although the high incidence of diarrhea and URI underscored the high stress levels of cats at this shelter, it was unfortunate that specific stress markers such as hormone levels, appetite, or body weight changes were unrecorded.

A standardized treatment protocol at animal shelters to prevent and medicate commonly observed disease is clearly justified not only for animal welfare, but also for public health. The main recommendations from this study to improve cat health in shelter settings include: (1) establishing strategies to preemptively reduce stress are warranted, and include providing hiding boxes, separating cat housing from dog cages and runs, rese exposing cats to less environmental noise, reducing population density, and providing consistent care by trained personnel; and (2) avoid using corticosteroids, interferon, antihistamines, or multiple antibiotics in the face of diarrhea or URI. To be prepared for future disasters, an effective animal sheltering strategy is axiomatic in helping to secure the safety of animals and humans in a community.

## Supporting information

S1 File Data file. (XLSX)
